# On-Chip AC Electrothermal Pump for Pulsatile Perfusion

**DOI:** 10.3390/mi17040492

**Published:** 2026-04-17

**Authors:** Itaru Kawata, Sosuke Kobayashi, Yoshiyasu Ichikawa, Masahiro Motosuke

**Affiliations:** 1 Department of Mechanical Engineering, Graduate School of Engineering, Tokyo University of Science, 6-3-1, Niijuku, Katsushika-ku, Tokyo 125-8585, Japan; 2 Department of Mechanical Engineering, Faculty of Engineering, Tokyo University of Science, 6-3-1, Niijuku, Katsushika-ku, Tokyo 125-8585, Japan; 3 Water Frontier Research Center, Research Institute for Science and Technology, Tokyo University of Science, 1-3, Kagurazaka, Shinjuku-ku, Tokyo 162-8601, Japan

**Keywords:** microphysiological system (MPS), pulsatile flow, electrothermal flow, mesenchymal stem cell (MSC), differentiation

## Abstract

Microphysiological systems (MPSs) have emerged as promising platforms for drug discovery and in vitro pharmacological testing. MPSs aid to reproduce physiologically relevant microenvironments, in which controlled perfusion can play important role. In this study, an on-chip AC electrothermal (ACET) pump was developed for pulsatile perfusion in microfluidic cell culture systems. The proposed pump generates fluid motion through the interaction between an applied electric field and temperature-dependent gradients in the electrical properties of the fluid. Pulsatile perfusion was produced by periodic application of an AC voltage to the electrode array, and the pulsation cycle could be controlled electrically. The maximum flow velocity increased with the applied AC voltage, demonstrating tunable flow generation by the ACET pump. To evaluate the applicability of the developed system to cell culture, human mesenchymal stem cells (hMSCs) were cultured under pulsatile perfusion conditions for five days. The results showed that osteogenic differentiation under pulsatile perfusion was higher than that under static culture conditions. These findings demonstrate the potential of the proposed on-chip ACET pump as a simple and effective platform for generating physiologically relevant pulsatile perfusion in microphysiological systems.

## 1. Introduction

Since the 1990s, the cost of new drug development has continued to increase, creating a strong demand for alternative testing methods that can reduce development costs [1,2]. In this context, microphysiological systems (MPSs) have attracted considerable attention as promising platforms for pharmacokinetic and drug screening studies [3]. An MPS is a device that recreates human organs or tissues in a microscale environment by culturing human cells in microfluidic channels. Compared with conventional static culture-based assays, MPS-based testing is expected to provide more physiologically relevant cellular functions and, consequently, more accurate evaluation of drug responses [4].

To improve the physiological relevance of MPSs, it is important to reproduce the physicochemical conditions of the in vivo environment as accurately as possible. In particular, recirculating flow is essential for mimicking the circulation of blood and interstitial fluids in the body, and therefore cell culture in MPSs should ideally be conducted under perfusion conditions with a circulation system [5]. The use of a recirculating system is also beneficial for reducing the consumption of culture medium and reagents in pharmacokinetic assays, thereby lowering experimental costs. In addition, blood flow in arteries is inherently pulsatile because of the periodic pumping action of the heart. Therefore, reproducing pulsatile flow in cell culture systems is expected to improve cellular activity and functionality compared with static or steady-flow conditions [6]. Mokhaled et al. exposed human aortic endothelial cells (HAECs) to steady and pulsatile flow and showed that the orientation of actin filaments involved in intracellular transport differed depending on the flow condition [7]. Josephine et al. also demonstrated that perfusion culture of human mesenchymal stem cells (hMSCs) under pulsatile flow using a peristaltic pump significantly promoted cell proliferation compared with static culture [8]. These studies suggest that pulsatile flow can provide a distinct mechanical microenvironment from steady flow and static culture, thereby enhancing cellular activity and function. Accordingly, the use of a pump capable of generating pulsatile flow in a recirculating MPS platform is expected to improve the physiological relevance of in vitro culture systems [9].

At present, syringe pumps and peristaltic pumps are among the most commonly used pumping systems [10]. Because syringe pumps transport fluid by driving a plunger at a constant speed, they can provide stable flow at a constant flow rate. However, the pumped fluid is typically discarded as waste, resulting in increased consumption of medium and reagents [11]. In addition, syringe pumps require tubing to connect the pump to the microfluidic device, which may cause practical problems such as bubble formation and fluid leakage [12]. For this reason, gravity-driven pumps, which are tubeless and mechanically simple, are also widely used [13]. Gravity-driven systems generate flow from the hydrostatic pressure difference between reservoirs at different heights and thus offer advantages such as low cost and simple operation [14]. On the other hand, the flow rate changes as the liquid level difference varies, making it difficult to maintain a continuous and stable flow [15]. These limitations highlight the need for a new pumping mechanism for MPS applications that differs from conventional systems while still providing stable fluid transport.

**Figure 1:**
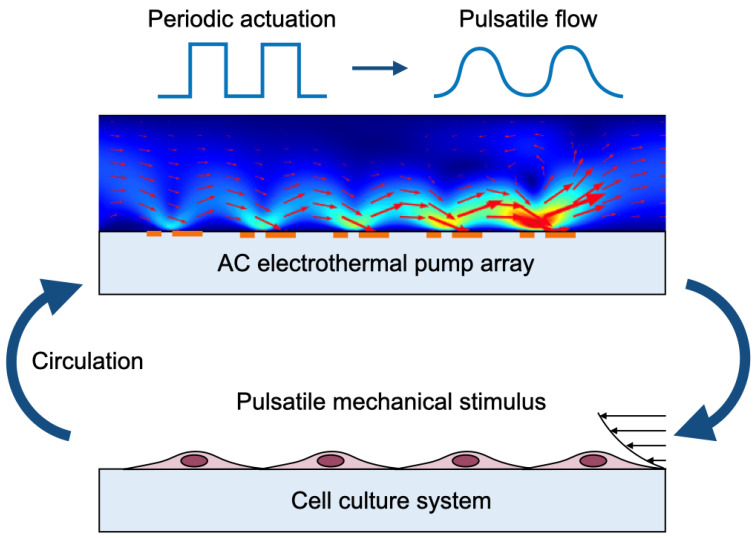
On-chip AC electrothermal pump for pulsatile perfusion.

In this study, we focused on an electric-field-driven pump based on electrothermal (ET) flow. ET flow is an electrohydrodynamic phenomenon induced by a nonuniform electric field in a conductive liquid and has been used for applications such as microparticle manipulation and micromixing [16]. The driving force of ET flow depends on the interaction between the nonuniform electric field and temperature gradients generated by Joule heating during operation. Because many biological fluids surrounding cells, such as plasma and tissue fluids, are highly conductive, temperature gradients can be readily generated in such media [17]. Therefore, ET flow is a promising mechanism for fluid transport in MPSs. In addition, an ET pump can generate flow by integrating electrodes beneath the microchannel, enabling a simple on-chip pumping structure without external mechanical actuators and facilitating integration into recirculating microfluidic systems. Lang et al. demonstrated an AC electrothermal circulatory pumping chip for cell culture and showed that ET-driven on-chip pumping can support long-term cell culture in a compact microfluidic platform [18]. Their study highlighted the feasibility of continuous circulatory pumping using AC electrothermal flow. In contrast, the present study focuses on the generation of pulsatile perfusion by temporally modulating the applied AC voltage and on evaluating its effect on osteogenic differentiation of human mesenchymal stem cells (hMSCs). Based on this concept, we developed a cell culture device equipped with an electric-field-driven pump capable of generating pulsatile flow, as shown in Figure 1. To demonstrate the usefulness of the proposed device, osteogenic differentiation of human mesenchymal stem cells (hMSCs) was performed in the device under pulsatile perfusion conditions.

## 2. Materials and Methods

### 2.1. Cell Culture Device

#### 2.1.1. AC Electrothermal Pump

The electric-field-driven pump used in this study operates based on electrothermal (ET) flow. ET flow is generated by the interaction between a nonuniform electric field and gradients in the electrical properties of a conductive fluid. To induce ET flow, electrodes must first be integrated on the bottom surface of the microchannel.

When an AC voltage is applied to the electrodes placed on the channel bottom, Joule heating generates a temperature field described by Equation (1):

(1) $$\nabla^{2}T=-\frac{\sigma {\left|E\right|}^{2}}{\lambda }$$

where *T* is temperature, ***E*** is the applied electric field, *λ* is the thermal conductivity of the fluid, and *σ* is the electrical conductivity of the fluid.

Because the electrical properties of the fluid, such as the electrical conductivity *σ* and permittivity *ε*, depend on temperature, the temperature field given by Equation (1) produces spatial gradients in these properties. The interaction between these gradients and the nonuniform electric field generates an electrical body force in the fluid. This electrical force consists of Coulomb and dielectric components and can be expressed by Equation (2) using *E*, *σ*, *ε*, the angular frequency of the applied AC voltage *ω*, and the charge relaxation time *τ* (=*ε*/σ).

(2) $$F_{ET}=-\frac{1}{2}\left[\left(\frac{\nabla \sigma }{\sigma }-\frac{\nabla \varepsilon }{\varepsilon }\right)E\frac{\varepsilon E}{1+{\left(\omega \tau \right)}^{2}}+\frac{{\left|E\right|}^{2}\nabla \varepsilon }{2}\right]$$

And overall velocity field, ***u***, of the fluid with the effect of the electrothermal body force ***F****_ET_* can be obtained from the Navier–Stokes equation with low Reynolds number assumption, namely Stokes equation, together with the continuity equation:

(3) $$-\nabla p+\eta \nabla^{2}u+F_{ET}=0$$

(4) ∇u=0

where *p* is the pressure and *η* is the fluid viscosity.

According to the theory [19], the driving force of ET flow is proportional to the square of the electric field and the square of the temperature gradient. Since Joule heating is proportional to |***E***|^2^, the ET flow velocity is expected to be proportional to the fourth power of the applied voltage. This relationship indicates that the flow generated by the pump can be controlled by modulating the applied electric field.

**Figure 2:**
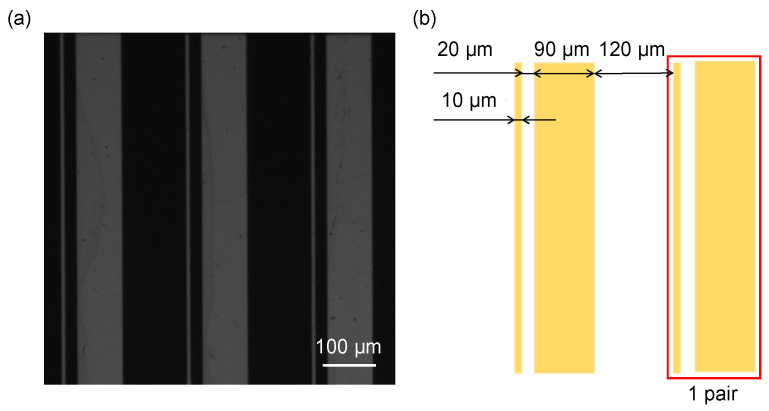
(**a**) Image and (**b**) geometry of the electrode.

**Figure 3:**
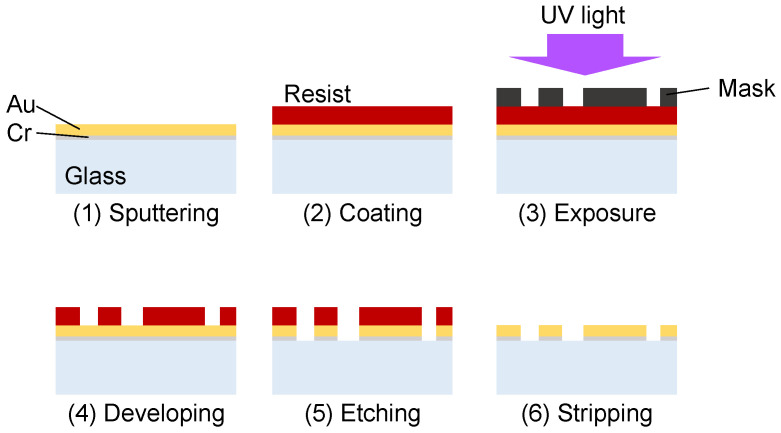
Fabrication process of electrode array.

The electric-field-driven pump was fabricated by patterning an asymmetric Au electrode array on a glass substrate. The concept of an electric-field-driven micropump using an asymmetric electrode array was first proposed by Ajdari in the context of AC electroosmotic flow [20] and was later extended to AC electrothermal (ACET) flow by Wu et al. [21]. The electrode geometry and dimensions are shown in Figure 2. The array consisted of 30 pairs of asymmetric electrodes with widths of 10 μm and 90 μm connected in series. The gap between adjacent electrodes in each pair was 20 μm, and the electrode pairs are aligned along the channel with the intervals of 120 μm. These electrode pairs were electrically connected so that AC voltage was imposed between narrow and wide electrodes of the array. The electrodes were fabricated by depositing Au on a glass substrate by sputtering (CFS-4EP-LL, Shibaura Mechatronics, Yokohama, Japan), transferring a photoresist mask by photolithography, and patterning the electrode structure by wet etching (Figure 3) [22]. A thin Cr adhesion layer was inserted between the glass substrate and the Au layer. The Cr and Au thicknesses were 10 nm and 80 nm, respectively.

#### 2.1.2. Microfluidic System

**Figure 4:**
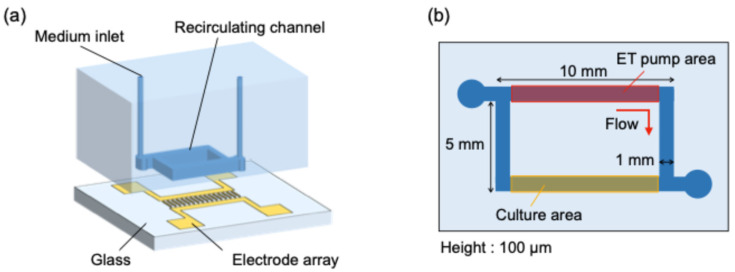
Schematics of (**a**) a cell culture device and (**b**) channel geometry.

A schematic illustration of the device used in this study, together with the microchannel geometry, is shown in Figure 4. The cell culture device consisted of a glass substrate equipped with the electrode array for the electrothermal pump and a rectangular polydimethylsiloxane (PDMS) microchannel with a width of 1 mm and a height of 100 μm. The microchannel was composed of two 10 mm-long sections (ET pump and cell culture) and two 5 mm-long rectangular sections connected in series. The ET pump generates pulsatile flow in the pumping region, which is transmitted to the culture region through the microchannel network, thereby establishing a recirculating pulsatile perfusion in the device.

The pumping region and the culture region were separated with 5 mm distance and arranged in parallel in order to reduce thermal interference from the pump and to maintain a more stable culture environment in the cell culture section.

### 2.2. Evaluation of Device Performance

#### 2.2.1. Flow Characterization of the AC Electrothermal Pump

To evaluate the flow generated in the culture region during operation of the AC electrothermal pump, flow visualization was performed using fluorescent tracer particles. The working fluid used in the flow characterization experiments was mesenchymal stem cell osteogenic differentiation medium (ODM, PromoCell, Heidelberg, Germany), which was also used for osteogenic differentiation of human mesenchymal stem cells (hMSCs). Fluorescent particles with a diameter of 1 μm (FluoSpheres, Thermo Fisher Scientific, Waltham, MA, USA) were added to the medium as tracer particles.

The experiments were conducted under temperature-controlled conditions to mimic the cell culture environment. The culture device was maintained at 37 °C using a glass heater (Tokai Hit, Sizuoka, Japan). The AC electrothermal pump was actuated by applying an AC voltage, generated by a function generator, to the electrodes integrated on the bottom surface of the microchannel. To induce pulsatile flow, the AC voltage was applied periodically with burst modulation. The applied AC voltage ranged from 7 to 9 Vpp, the frequency was fixed at 1 MHz, the actuation period was 2 s, and the duty ratio was 0.5. The tracer motion was recorded at 80 fps using an inverted microscope with a 20× objective lens. The recorded images were analyzed by micro-particle image velocimetry (micro-PIV) [23] to determine the flow velocity in the culture region.

#### 2.2.2. Numerical Evaluation of Temperature Distribution During Pump Operation

During operation of the AC electrothermal pump, a temperature rise is generated by Joule heating. Because Joule heating generated by the applied AC electric field may affect cell viability and differentiation, it is important to confirm that the temperature rise in the culture region remains within an acceptable range. Therefore, the temperature distribution in the culture region during pump operation was evaluated numerically.

The numerical analysis was performed using a finite element solver (COMSOL Multiphysics^®^ v6.3). A three-dimensional model of the culture device was constructed, in which the microchannel was filled with a fluid representing the culture medium. In the actual device, the electrode thickness was 90 nm, which is sufficiently thinner than the channel height (100 μm). Therefore, the electrode thickness was neglected in the present simulation. The electrical conductivity of the working fluid was set to that of MEM medium, which is widely used for human cell culture, i.e., 1.4 S/m. The other thermal properties of the fluid were assumed to be identical to those of water. The numerical model for AC electrothermal (ACET) flow was validated in our previous studies [24,25].

As electrical boundary conditions, electric potentials were applied to the upstream and downstream electrodes of each electrode pair. The potentials of the upstream and downstream electrodes are given by Equations (5) and (6), respectively.

(5) $$V_{up}=V_{rms}=\frac{V_{pp}}{2\sqrt{2}}$$

(6) $$V_{down}=-V_{rms}=-\frac{V_{pp}}{2\sqrt{2}}$$

Here, *V*_rms_ denotes the root-mean-square voltage, and *V*_pp_ is the peak-to-peak voltage. The electric field ***E*** was calculated from the electric potential distribution using Equations (7) and (8). The applied voltage ranged from 7 to 9 V_pp_, and the AC frequency was fixed at 1 MHz.

(7) $$\nabla^{2}V=\frac{\sigma }{\varepsilon }$$

(8) E=−∇V

The temperature field was calculated using the steady-state heat conduction equation, given by Equation (9). Here, the volumetric heat generation due to Joule heating is considered. For the thermal boundary conditions, the PDMS and glass walls were assumed to be isothermal at 37 °C.

(9) $$\lambda \nabla^{2}T+\sigma {\left|E\right|}^{2}=0$$

It should be noted that the present temperature evaluation is based solely on numerical simulation and was not directly validated by experimental temperature measurements in the microchannel. Therefore, the simulation results should be interpreted as a design-oriented estimation of the temperature rise during pump operation.

#### 2.2.3. Evaluation of Osteogenic Differentiation Induced in hMSCs

To evaluate the applicability of the fabricated device to cell culture, osteogenic differentiation of human mesenchymal stem cells (hMSCs) was performed in the microchannel. Surface coating of the glass substrate with an adhesion-promoting factor was performed to ensure stable cell attachment. In this study, fibronectin derived from bovine plasma (PromoCell) was used as the coating material. Fibronectin was diluted in phosphate-buffered saline (PBS) to a concentration of 1 μg/mL and introduced into the channel.

Before cell seeding, ethanol was perfused through the microchannel to wash the channel and remove trapped air bubbles. The fibronectin solution was then introduced to fill the channel. After incubation under a clean bench for 30–60 min, the channel was filled with Mesenchymal Stem Cell Growth Medium 2 (GM, PromoCell) to remove the excess coating solution. A cell suspension prepared in GM at a concentration of 1.0 × 10^6^ cells/mL was then introduced into the channel to seed the cells. After seeding, the device was placed in an incubator under standard culture conditions (37 °C, 95% humidity, and 5% CO_2_) for 12–24 h to allow the cells to attach to the substrate.

After cell attachment, the culture medium in the channel was replaced from GM to osteogenic differentiation medium (ODM, PromoCell). The electrothermal pump was then operated at 8 Vpp and 1 MHz with an actuation period of 2 s, and the hMSCs were exposed to pulsatile flow for 5 days. During the differentiation experiment, ODM was continuously supplied at 2 μL/h using a syringe pump to prevent cell death caused by nutrient depletion. Because the flow velocity generated by the syringe pump was much smaller than that induced by the electrothermal pump, the mechanical stimulation applied to the cells was expected to be dominated by the pulsatile flow generated by the AC electrothermal pump.

After 5 days of culture, alkaline phosphatase (ALP), an enzymatic marker of osteogenic differentiation, was used for cell staining. Prior to ALP staining, the cells were fixed on the glass substrate by filling the channel with a cell fixation solution (Takara Bio, Kusatsu, Japan) containing acetone and ethanol. Subsequently, an alkaline phosphatase premix substrate solution (Takara Bio), prepared by dissolving the reagent in sterile distilled water, was introduced into the channel. The device was then incubated for 15–45 min to allow staining of differentiated cells.

## 3. Results and Discussion

### 3.1. Pulsatile Flow Measurement

**Figure 5:**
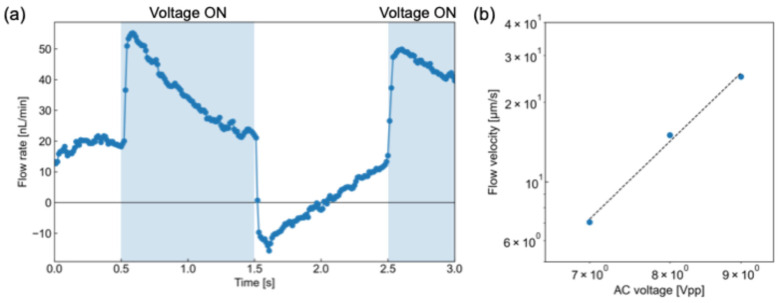
Characterization of pulsatile flow generated by the AC electrothermal pump in the culture region, showing (**a**) the time variation in the flow rate at the culture area under 8 Vpp and (**b**) the log–log relationship between the applied AC voltage and the maximum flow velocity.

Figure 5a shows the time-dependent variation in the flow rate in the culture region, based on the PIV results, at an applied voltage of 8 Vpp. As shown in the Figure, pulsatile flow was successfully generated in the culture region. The conversion from centerline velocity to volumetric flow rate was based on the theoretical velocity distribution for fully developed laminar flow in a rectangular channel [26]. It was also observed that backflow occurred in the culture region during the voltage-off period of the actuation cycle (i.e., 0 Vpp). The tracer particles moved downstream overall while exhibiting periodic pulsatile motion (see Video S1). During the voltage-on period, the AC electrothermal pump actively drives the fluid in the downstream direction. In contrast, during the voltage-off period, the driving force disappears and partial backflow occurs because of pressure redistribution and hydraulic relaxation in the closed microchannel network. Nevertheless, the forward transport during the voltage-on period is greater than the reverse transport during the voltage-off period, resulting in net downstream flow over one actuation cycle. Therefore, this result indicates that the flow generated by the electrothermal pump provided net unidirectional transport despite the presence of transient backflow. Therefore, medium and reagent exchange in the culture region can be achieved by the AC electrothermal pulsation. In addition, the pulsation period of the flow was consistent with the 2 s voltage actuation cycle, in which the AC voltage was periodically switched on and off, indicating that the pulsation cycle can be controlled by adjusting the temporal pattern of the applied voltage. No obvious bubble generation was observed under the present operating conditions. This is likely because AC electrothermal flow is typically operated at sufficiently high AC frequencies, which reduce Faradaic reactions and help minimize electrolysis at the electrode interface [27]. These results suggest that the proposed AC electrothermal pump can reproduce periodically modulated flow conditions relevant to physiological environments in microfluidic devices. The shear stress applied to the cells was estimated to be approximately 7 mPa. This level is much smaller than typical vascular shear stress, but falls within the low-shear regime relevant to interstitial-flow-level mechanical stimulation for MSCs [28]. Therefore, the pulsatile flow generated in the present device is expected to provide a weak but biologically relevant mechanical stimulus to the cultured hMSCs.

Figure 5b presents the relationship between the applied AC voltage and the maximum velocity of the pulsatile flow in the culture region in a log–log representation. The maximum flow velocity increased as the applied voltage increased. Because Joule heating is enhanced at higher applied voltages, the resulting temperature gradient becomes larger, leading to an increase in the driving force of ET flow. Since the ET driving force is theoretically expected to be proportional to the fourth power of the applied voltage, the voltage dependence of the measured velocity was evaluated in terms of a power-law relationship. The data showed an approximately linear trend on the log–log plot with a slope close to 4, indicating that the measured flow was governed primarily by ET flow. These results confirm that the proposed device can generate pulsatile flow in the culture region based on the ET pumping mechanism.

### 3.2. Temperature Evaluation

**Figure 6:**
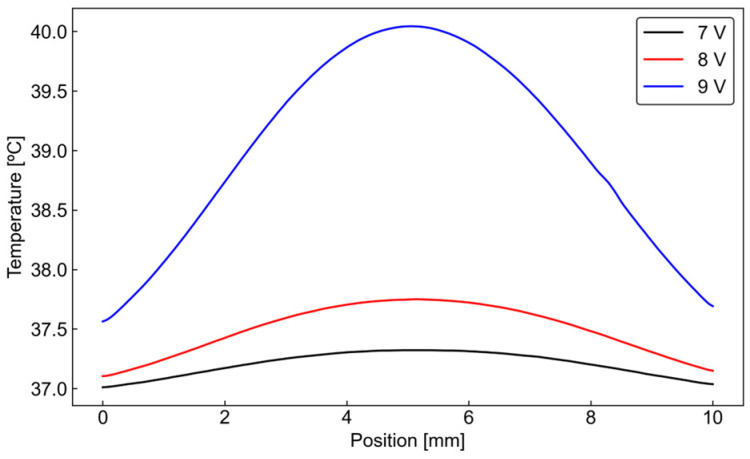
Temperature distribution at the culture channel at different applied voltages obtained by numerical simulation.

Figure 6 shows the temperature distribution in the culture region at different applied voltages. As the applied voltage increased, the temperature in the culture region also increased. In particular, at 9 Vpp, the maximum temperature rise in the culture region exceeded 3 °C, indicating that the thermal effect caused by Joule heating may become excessive under this condition. In contrast, at lower applied voltages, the temperature rise in the culture region was more limited. Note that the effect of the pump-induced flow on the temperature field is negligible here because of the low-Peclet-number nature of the flow. In other words, the temperature field is governed primarily by heat conduction within the device, rather than by heat transport through the liquid flowing in the channel.

In general, human cells function most stably around 37 °C, and physiological thermoregulation maintains body temperature within approximately ±1–2 °C of this value [29]. Therefore, in a cell culture device, the temperature variation in the culture region should preferably remain within a similar range. From this viewpoint, operation at 9 Vpp may impose an undesirably large thermal load on the cultured cells, whereas lower applied voltages are more suitable for cell culture because the temperature rise can be kept relatively small. Accordingly, operation at 8 Vpp or less is considered preferable for cell culture experiments in the present device.

### 3.3. Osteogenic Differentiation of hMSCs

**Figure 7:**
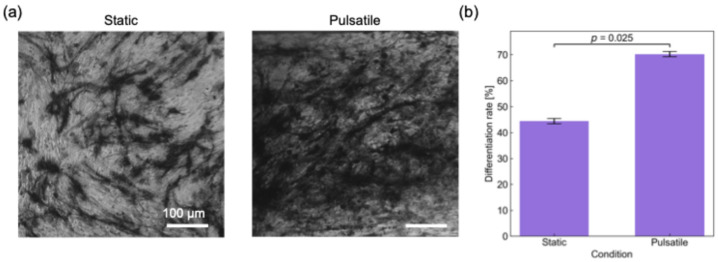
Osteogenic differentiation of hMSCs cultured in the proposed device, showing (**a**) representative ALP-stained images under static conditions (**left**) and pulsatile-flow conditions (**right**), where stained regions are shown in black and unstained regions in white, and (**b**) the quantified differentiation rate under each condition.

hMSCs were cultured in the device according to the procedure described in Section 2.2.3. Figure 7a shows representative images of ALP-stained cells after culture under static and pulsatile-flow conditions, where the left panel corresponds to the static condition and the right panel corresponds to the pulsatile-flow condition. In these images, stained regions are shown in black and unstained regions in white. The stained regions indicate the presence of alkaline phosphatase (ALP), which is an enzymatic marker of osteogenic differentiation, and therefore correspond to differentiated osteoblast-like cells [30]. As shown in Figure 7a, a portion of the cells was stained under both conditions, indicating that osteogenic differentiation of hMSCs progressed in both the static and pulsatile-flow cultures. In addition, the overall morphology of the hMSCs and differentiated cells under pulsatile-flow conditions was similar to that observed under the static condition, suggesting that the pulsatile flow generated by the electric-field-driven pump did not cause any obvious adverse morphological changes in the cells.

The differentiation rate of hMSCs under each condition was then quantified from the images shown in Figure 7a. The differentiation rate was defined as the ratio of the stained cell area to the total cell-occupied area in the image and was calculated by image processing after extraction of the stained regions. The resulting differentiation rates are shown in Figure 7b. In addition, the Mann–Whitney U test [31] was performed to compare the differentiation results between the two conditions. As shown in Figure 7b, the average differentiation rate under pulsatile-flow conditions was approximately 1.5 times higher than that under the static condition. The calculated *p*-value was smaller than the significance level of 0.05, indicating that the difference between the pulsatile-flow and static conditions was statistically significant.

This result suggests that the pulsatile flow generated by the electric-field-driven pump was associated with the enhanced osteogenic differentiation of hMSCs. A possible reason is that the fluid-mechanical stimulation provided by the pulsatile flow enhanced mechanotransduction-related processes involved in osteogenic differentiation. This tendency is consistent with a previous study [28], who reported that hMSC differentiation under flow stimulation resulted in a significant increase in ALP expression compared with static culture. Therefore, the present results indicate that pulsatile flow generated by the proposed electric-field-driven pump can influence and enhance the osteogenic differentiation of hMSCs. However, because additional biological controls were not included in the present study, the result should be interpreted as an initial demonstration of the potential effect of pulsatile perfusion on osteogenic differentiation. Also, a more detailed analysis of the influence of pulsatile waveform on cell behavior will be an important subject of future study.

## 4. Conclusions

In this study, a cell culture device integrated with an on-chip AC electrothermal pump was developed, and its applicability to pulsatile perfusion and osteogenic differentiation of human mesenchymal stem cells (hMSCs) was investigated. By periodically modulating the applied AC voltage, pulsatile ET flow with net downstream transport with partial backflow was generated in the culture region of the device. The pulsation period of the induced flow was consistent with the voltage actuation cycle, indicating that the flow pattern can be controlled electrically by the applied AC voltage.

Numerical analysis of the temperature distribution showed that the temperature rise in the culture region increased with increasing applied voltage. In particular, operation at 9 Vpp caused a temperature increase exceeding 3 °C in the culture region, suggesting that the thermal effect may become excessive for cell culture under this condition. In contrast, lower applied voltages produced a smaller temperature rise, indicating that operation 8 Vpp or less is preferable for cell culture applications in the present device.

Under pulsatile-flow conditions generated by the proposed pump, osteogenic differentiation of hMSCs was evaluated and compared with that under static culture conditions. The differentiation rate under pulsatile flow was significantly higher than that under the static condition, demonstrating that flow conditions affected the differentiation outcome. These results indicate that pulsatile flow generated by the proposed AC electrothermal pump can affect the osteogenic differentiation of hMSCs and suggest that the device is a promising platform for introducing mechanically relevant perfusion into microphysiological cell culture systems.

## Data Availability

The data presented in this study are available from the corresponding author upon reasonable request.

## Supplementary Materials

The following supporting information can be downloaded at: https://www.mdpi.com/article/10.3390/mi17040492/s1. Video S1: Pulsatile movement of tracer particles

## Abbreviations

The following abbreviations are used in this manuscript:

AC	Alternating current
ALP	Alkaline phosphatase
ET	Electrothermal
MSC	Mesenchymal stem cell
